# Renal Medullary Carcinoma: A Call for Screening and Early Diagnosis

**DOI:** 10.7759/cureus.96915

**Published:** 2025-11-15

**Authors:** Harikrishna Choudary Ponnam, Pallavi Shirsat, Saketh Parsi, Usha Ravi, Kunal Sonavane, Keyvan Ravakhah

**Affiliations:** 1 Internal Medicine, Summa Health, Akron, USA; 2 Nephrology, Minden Medical Center, Minden, USA; 3 Hospital Medicine, Ascension Seton Medical Center, Austin, USA; 4 Pediatrics, Tulare Pediatric Group, Tulare, USA; 5 Internal Medicine, Willis Knighton Medical Center, Bossier City, USA; 6 Internal Medicine, Mount Carmel Medical Center, Columbus, USA; 7 Internal Medicine, St. Vincent Charity Medical Center, Cleveland, USA

**Keywords:** aggressive cancer, gross hematuria, palliative and supportive care, renal medullary carcinoma, sickle cell trait

## Abstract

Renal medullary carcinoma (RMC) is a highly aggressive malignancy associated strongly with sickle cell trait (SCT). This primarily affects young individuals, often presenting with nonspecific symptoms that cause delayed diagnosis, such as hematuria, flank pain, and weight loss, leading to a poor prognosis.

We describe the case of a young adult man with no significant past medical history who presented to the emergency department with sudden-onset abdominal pain. Initial workup revealed mild anemia and microscopic hematuria. Contrast-enhanced computed tomography identified a right renal mass and retroperitoneal lymphadenopathy suggestive of metastatic disease. Renal biopsy confirmed RMC. Due to the advanced stage of disease and limited treatment options, the patient and his family opted for palliative care. He passed away at home several weeks later under hospice services. This case highlights the importance of considering RMC in young patients with SCT who present with unexplained abdominal pain and hematuria. Early recognition, multidisciplinary evaluation, and emphasis on supportive care are critical components in the management of this rare and aggressive malignancy.

## Introduction

Renal medullary carcinoma (RMC) is a rare and highly aggressive form of renal cancer, almost exclusively affecting young Black/African individuals with sickle cell trait (SCT) [1]. Despite accounting for less than 1% of all renal tumors [2], RMC is notable for its poor prognosis, with most cases presenting at an advanced or metastatic stage and a median survival of only 13.8 ± 3 months. The average age at diagnosis is 28.0 years, with a standard deviation of ±12.0. The disease disproportionately affects men (76%) compared to women (24%), and Black patients with RMC are significantly less likely to receive surgical intervention, a fivefold higher risk of death compared to White patients [3].

Originating from the renal medulla, RMC is typically resistant to conventional chemotherapy, contributing to poor clinical outcomes. The diagnosis is usually delayed. Patients commonly present with macroscopic hematuria, and standard treatment options, such as chemotherapy, radiation, and targeted therapies used in other renal cancers, have demonstrated limited effectiveness in RMC cases [4]. Additionally, recent studies have identified a potential link between high-intensity physical activity and an increased risk of RMC in SCT carriers, possibly due to renal medullary hypoxia induced by strenuous exercise [5].

Currently, the standard approach to managing RMC involves nephrectomy followed by platinum-based chemotherapy. Some promising outcomes have been reported with the addition of bortezomib, including occasional complete responses [6].

## Case presentation

A previously healthy young adult man presented to the emergency department with acute-onset right-sided abdominal pain, nine out of 10 in intensity, radiating to the flank with no aggravating or relieving factors. He denied trauma, fever, dysuria, hematuria, weight loss, or any history of chronic illness. Vitals were as follows: Blood pressure 118/60 mm Hg, heart rate 72 bpm, respiratory rate 20 per minute, and temperature 37 degrees. On inspection, there was no visible swelling, erythema, or other skin changes over the right flank. Palpation revealed localized tenderness over the right flank without evidence of rebound tenderness or guarding, and costovertebral angle (CVA) tenderness was absent. No bruits or abnormal vascular sounds were auscultated over the flank region.

Laboratory findings revealed mild normocytic anemia on complete blood count, and the metabolic panel was within normal limits. Urinalysis showed microscopic hematuria in the absence of pyuria or proteinuria.

**Figure 1:**
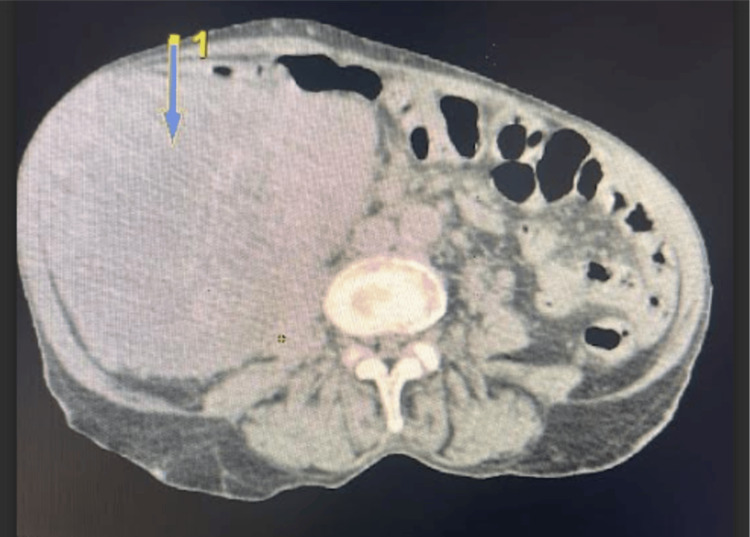
CT Scan Showing Right-Sided Renal Cell Carcinoma Showing Mass Effect on the Liver

A contrast-enhanced computed tomography (CT) scan of the abdomen and pelvis for pain revealed a heterogeneously enhancing mass in the right kidney (Figure 1). The patient underwent ultrasound-guided percutaneous renal biopsy, which revealed poorly differentiated carcinoma with histologic features consistent with RMC. Hemoglobin electrophoresis confirmed the presence of sickle cell trait (HBAS).

Given the presence of metastatic disease and the aggressive nature of RMC, the case was reviewed at a multidisciplinary tumor board, including oncology, nephrology, urology, radiology, and palliative care teams, due to the patient’s uncontrolled pain, significant weight loss, poor nutritional status, and multi-organ failure. Given the rapid progression of his illness and limited benefit from available systemic therapies, he and his family elected for comfort-focused care. They transitioned to home hospice in alignment with his goals and values.

He received supportive palliative measures aimed at managing symptoms and maintaining quality of life, including adequate pain control and emotional support. The patient passed away peacefully at home several weeks later.

## Discussion

RMC is an exceptionally aggressive and rare malignancy that predominantly affects young individuals, particularly those with SCT [3]. Its differential diagnoses include collecting duct carcinoma, urothelial carcinoma of the renal pelvis, and other high-grade renal neoplasms. This case highlights the importance of clinical awareness, molecular understanding, and public health measures in addressing this lethal disease. It also reinforces the urgent need for early detection, especially in high-risk populations such as adolescents and young adults with SCT.

In a multicenter retrospective cohort study of 34 patients, the median age at diagnosis was 19 years, reflecting the disproportionate burden of RMC on younger populations and highlighting the necessity for clinicians to maintain a high index of suspicion in patients presenting with unexplained flank pain, hematuria, or renal masses [7].

Importantly, this case illustrates a common clinical gap. The patient was unaware of their SCT status until after the diagnosis of RMC. Although SCT is routinely screened for in U.S. newborns, this information is often lost in transition to adult healthcare. According to the 2024 CDC guidelines, there is an increased emphasis on SCT identification during reproductive years and improved education on rare but serious complications such as RMC. Early interventions, such as routine urine dipstick screening for microscopic hematuria in high-risk individuals, could facilitate earlier detection and potentially improve outcomes before metastatic progression occurs [4].

At the molecular level, RMC is most notably characterized by the loss of SMARCB1 (sucrose non-fermentable matrix-associated, actin-dependent regulator of chromatin subfamily B member 1), a tumor suppressor gene [8]. The inactivation of SMARCB1 not only serves as a diagnostic hallmark but also reveals potential therapeutic vulnerabilities. Recent advances, including next-generation sequencing and the development of patient-derived xenograft models, have provided crucial insights into RMC biology and enabled preclinical testing of targeted agents. Despite these advances, translation into durable clinical outcomes remains limited [8].

Most patients with RMC present with metastatic disease at diagnosis. Upfront radical nephrectomy may be appropriate for individuals with good performance status and limited metastatic burden, or following a favorable response to systemic therapy. Currently, cytotoxic platinum-based chemotherapy provides the most effective, though typically short-lived, palliative benefit [9]. However, the prognosis remains bad even with aggressive multimodal therapy. Most systemic therapies, including targeted agents and immunotherapies, have shown minimal efficacy. Consequently, early integration of palliative care is essential to ensure symptom control and maintain quality of life throughout treatment.

Emerging data suggest that CA-125 may serve as a promising serum biomarker for disease burden in RMC [5]. If validated in larger cohorts, this marker could play a significant role in non-invasive disease monitoring, potentially aiding in both earlier diagnosis, and real-time assessment of therapeutic response.

Given the aggressive nature and high recurrence rate of RMC-even during postoperative recovery-upfront systemic chemotherapy should be strongly considered even for patients with localized disease [10]. This approach may help address micro metastatic disease early and improve progression-free survival. A defining hallmark of RMC is its rapid progression and poor prognosis, with median survival often reported as less than one year [11].

Finally, this case has clear public health implications. The demonstrated link between SCT and RMC should guide updates to routine screening protocols and inform genetic counseling strategies in high-risk communities. RMC occurs almost exclusively in people with SCT, existing screening programs for SCT could be expanded to include education on RMC risk and early renal symptom awareness. Rather than broad RMC screening, the focus should be on identifying SCT carriers and providing genetic counseling and symptom-based monitoring in high-risk groups. Proactive identification of SCT carriers can facilitate patient education, surveillance, and earlier clinical intervention, potentially altering the natural history of this devastating disease.

## Conclusions

RMC is a rare but devastating malignancy primarily affecting individuals with SCT. This case emphasizes the importance of early recognition, especially in young patients presenting with flank pain and hematuria. Renal ultrasound is recommended for high-risk individuals, including those with SCT or disease, African ancestry, a family history of RMC, or patients presenting with hematuria or unexplained abdominal or flank pain. Asymptomatic high-risk individuals should undergo annual ultrasound, while symptomatic patients should receive immediate imaging. Any suspicious lesion detected on ultrasound should be further evaluated with contrast-enhanced CT or MRI for confirmation and staging. Timely imaging, histologic confirmation, and interdisciplinary care are essential. In cases of advanced disease, early involvement of palliative care can significantly enhance patient comfort and facilitate effective end-of-life care planning. Identifying SCT remains important not for immediate therapy, but for risk identification, genetic counseling, and early recognition of RMC in at-risk individuals.
